# The Capability of Fiber Bragg Grating Sensors to Measure Amputees' Trans-Tibial Stump/Socket Interface Pressures

**DOI:** 10.3390/s130810348

**Published:** 2013-08-12

**Authors:** Ebrahim A. Al-Fakih, Noor Azuan Abu Osman, Arezoo Eshraghi, Faisal Rafiq Mahamd Adikan

**Affiliations:** 1 Center for Applied Biomechanics, Department of Biomedical Engineering, Faculty of Engineering, University of Malaya, Kuala Lumpur 50603, Malaysia; E-Mails: azuan@um.edu.my (N.A.A.O.); arezooeshraghi@yahoo.ca (A.E.); 2 Photonics Research Group, Department of Electrical Engineering, Faculty of Engineering, University of Malaya, Kuala Lumpur 50603, Malaysia; E-Mail: rafiq@um.edu.my

**Keywords:** prosthetic socket, trans-tibial amputee, interface pressure, fiber Bragg grating sensor

## Abstract

This study presents the first investigation into the capability of fiber Bragg grating (FBG) sensors to measure interface pressure between the stump and the prosthetic sockets of a trans-tibial amputee. FBG element(s) were recoated with and embedded in a thin layer of epoxy material to form a sensing pad, which was in turn embedded in a silicone polymer material to form a pressure sensor. The sensor was tested in real time by inserting a heavy-duty balloon into the socket and inflating it by using an air compressor. This test was conducted to examine the sensitivity and repeatability of the sensor when subjected to pressure from the stump of the trans-tibial amputee and to mimic the actual environment of the amputee's Patellar Tendon (PT) bar. The sensor exhibited a sensitivity of 127 pm/N and a maximum FSO hysteresis of around ∼0.09 in real-time operation. Very good reliability was achieved when the sensor was utilized for *in situ* measurements. This study may lead to smart FBG-based amputee stump/socket structures for pressure monitoring in amputee socket systems, which will result in better-designed prosthetic sockets that ensure improved patient satisfaction.

## 1. Introduction

Design of an improved Patellar Tendon Bearing (PTB) socket that prevents discomfort and provides satisfactory results requires a thorough knowledge of the interface pressure between the stump and trans-tibial PTB socket of an amputee. Despite considerable technological advancements in trans-tibial PTB socket design over the last few decades, many trans-tibial amputees still complain that their prosthesis causes a variety of complications such as edema, pressure ulcers, skin irritation, and dermatitis [1]. As a result, since the mid-1950s researchers have been involved in studies that pertain to these issues, proposing a variety of measurement systems such as electrical strain gauge (SG)-based transducers [2,3], F-socket transducer arrays (Tekscan Inc, Boston, MA, USA) [4,5], and finite element analysis [6]. The outcomes of their measurements have contributed to improved socket design [7]. However, these systems have numerous limitations [7,8]. Despite the high accuracy and sensitivity of the SG-based method, a modified test socket needs to be fabricated and openings must be made in the socket wall to mount the SG pressure transducers. This process is laborious and difficult and may lead to altered socket shape and inaccurate pressure measurements. The F-Socket array involves very thin sensors placed *in situ* at stump/socket interface, enabling measurements to be made without the need of a modified socket, but these sensors does not account for shear stresses and may crinkle and fail. On the other hand, the FBG sensors have prominent advantages over conventional measurement systems, such as their small size, light weight, flexibility, inherent safety, multiplexing capabilities, electromagnetic interference immunity, high spatial resolution, and high accuracy and sensitivity, in particular, for monitoring dynamic strain [9–12].

The FBG is a periodic variation of the refractive index of a single-mode optical fiber [13]. If a broadband light is coupled into the FBG fiber, a narrow wavelength band, whose peak is called the Bragg wavelength λ_B_, is reflected back [14,15],which depends on the period Λ of the modulation of the refractive index along the fiber core and the effective refractive index n_eff_:λ_B_ = 2 n_eff_Λ. When the FBG is subjected to external perturbations such as strain or pressure, the peak wavelength that is reflected back is shifted accordingly [13,16]. The axial strain changes the length of the FBG, which changes the grating period, Λ, and induces a change in n_eff_, the effective grating index [13]. The Bragg wavelength λ_B_ is commonly determined by using optical spectrum analyzers (OSA). We hypothesize that FBG technology can detect the interface pressure between the amputee stump and the socket wall.

Recently, FBG sensors have been identified and utilized in a wide range of applications in aeronautics, the automotive industry, structure monitoring in civil engineering and undersea oil exploration [14]. Unfortunately, only minor attempts have been conducted to explore the potential applications of FBG technology in the field of biomechanics and rehabilitation [17]. Kanellos and his colleagues proposed that FBG sensors have the potential to be used for pressure measurements in human-machine interfaces such as amputee sockets [18]. However, they have not established its practicality for full-scale implementation. For instance, sensor/socket integration was not performed, and sensor performance under dynamic loads was not evaluated. In this study, we practically investigated the feasibility of the FBG sensing concept for measuring the interface pressure between the stump of a trans-tibial amputee and a PTB prosthetic socket. We focused on the PT bar area in particular because it is a pressure-tolerant area of the stump and carries the majority of the trans-tibial amputee's total body weight while the person wears the PTB prosthetic socket [19]. The basic design and working principles of the sensor are presented, along with the results of the preliminary tests.

## 2. Materials and Methods

### 2.1. PTB Socket Fabrication

PTB sockets are custom fabricated on a case-to-case basis [2]. Therefore, the fabrication process still relies largely on the techniques, skills, and experience of the prosthetist [7]. The FBG sensor fabricated in this study is to be placed at the PT bar area, so the measurements can be obtained without fabricating a modified test socket. A normal PTB socket was fabricated for this study.

The prosthetist must know the volume of the amputee's stump to produce the perfect design. The main steps in fabricating PTB sockets are the following: First, the negative mold of the stump is created by wrapping plaster of Paris (POP) around the soft tissue of the stump. The POP is left for a while so that the shape of the stump forms properly. Second, the positive mold of the stump is produced based on the shape of the negative cast. Third, the positive mold is modified or rectified to form the intended shape. Fourth, the socket liner, which is made of polyethylene material, is formed. The product is then laminated. The final step is socket finishing [2]. Further details about the manufacturing procedures are provided by Radcliffe [20].

### 2.2. FBG Fabrication Technique

**Figure 1.**
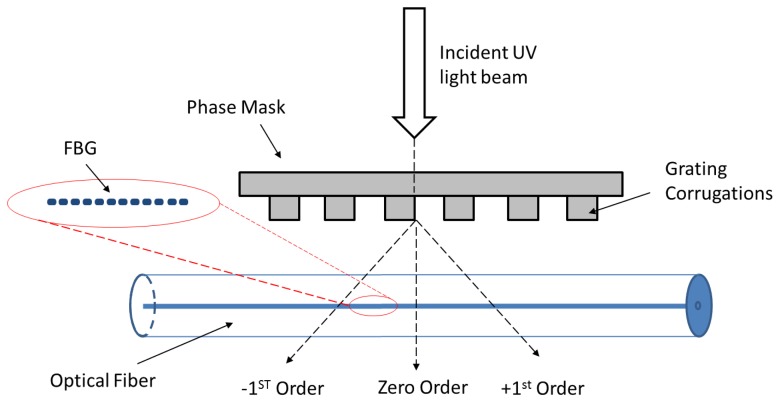
FBG fabrication process using Phase Mask Technique.

A uniform FBG was fabricated at Department of Physics, University of Malaya, using the Phase Mask technique as illustrated in Figure 1. The phase mask is made of silica glass as flat slab transparent to UV beams, whose shape of the periodic corrugations approximates a square wave in profile. The optical fiber is placed almost in contact with the corrugations of the phase mask and incident of UV lights in normal direction passes through the mask and then diffracts by those corrugations in the 0, +1, and −1 diffracted orders. The phase mask could be designed in a special way to suppress the diffraction into the zero order by controlling the depth of the corrugations in the phase mask. A 10 mm long FBG has been made for this study with a Bragg peak wavelength at around 1,550.952 nm. When embedding the FBG fiber in the host materials, this wavelength was shifted back to 1,550.825 nm due to the host material contraction upon curing.

### 2.3. Sensor Design and Fabrication

**Figure 2.**
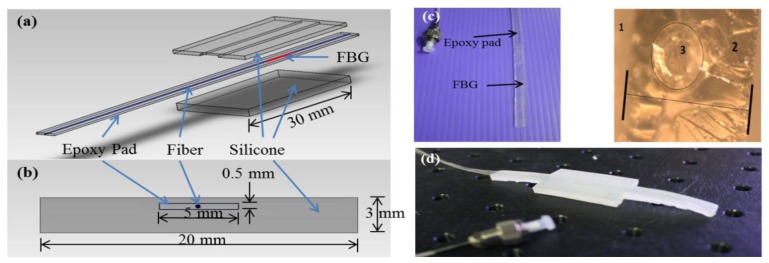
Structure of the pressure sensor. The epoxy pad (the strip-like pad shown in (**a**)) was placed between the upper (1 mm thick) and the lower (2 mm) sheets of silicone materials to form the pressure sensor. (**b**) The sensor cross-sectional area is 20 × 3 mm^2^ and the dimensions of the whole sensor were 30 × 20 × 3 mm^3^, and the sensitive surface area was 20 × 30 mm^2^. (**c**) Images of the epoxy pad and its microscopic cross-sectional area. (**d**) The image of the whole pressure sensor.

The application for which the FBG was used in this work required advanced protection due to the large pressure values at the PT bar that reach up to 230 kPa on average [2]. Therefore, epoxy (NOA 61, Norland Products Inc., Cranbury, NJ, USA) was used to protect the FBG fiber. A series of experiments was conducted to fabricate the typical epoxy pad to ensure that it can provide optimal protection for the FBG when subjected to the PT bar loads. The final dimension of this pad was 90 × 5 × 0.5 mm^3^ (Figure 2a,b). The epoxy pad was then placed between two sheets of silicone polymeric materials that form the pressure sensor shown in Figure 2.

### 2.4. Sensor Calibration

**Figure 3.**
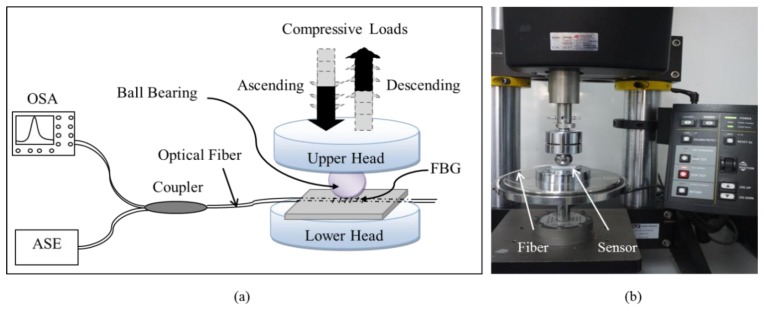
Schematic diagrams of the sensor calibration. (**a**) Simple schematic diagram illustrating the calibration experimental setup. (**b**) Image depicting the setup.

The sensor was calibrated by using an Instron Microtester 5,848 strain machine with a dynamic range of 30 N. As the initial contact of the PT onto the sensor surface is mostly pressure concentrated [3], we preferred to mimic this behavior by utilizing a ball bearing for the calibration to apply similar concentrated compressive loads onto the sensor (Figure 3). The machine was manually controlled, and its upper head speed was assigned a maximum of 0.5°mm/min. The sensor was placed on the lower head of the machine and optically connected to OSA. The sensor underwent increasing and decreasing compressive loads in seven experimental trials with a dynamic range starting from 0 N progressing up to 30° N before being gradually decreased in steps, starting from 30 N down to 0 N. The peak wavelength shifts reflected back by the FBG were recorded accordingly. Throughout the experiments, the room temperature was maintained at a constant level so the FBG would measure only the applied forces. Thus there was no need to isolate the FBG from the temperature variations.

### 2.5. Experiments

**Figure 4.**
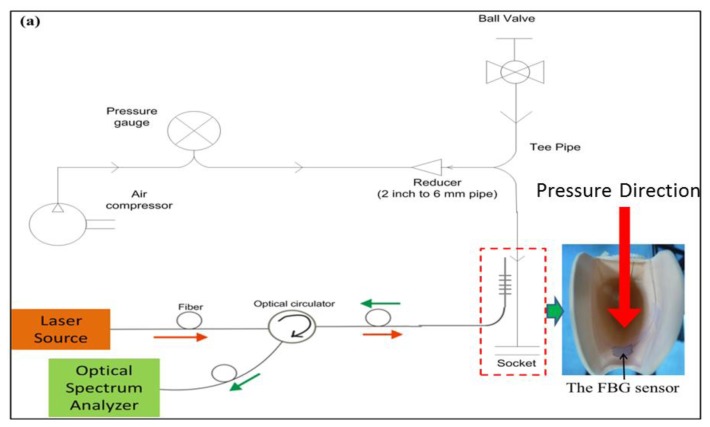

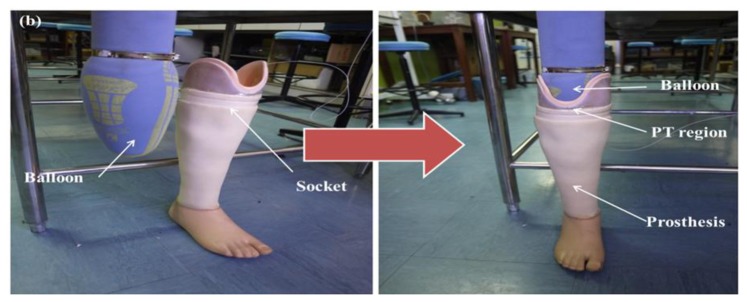
Experimental setup of the sensor functionality test. (**a**) A simple schematic diagram of the *in situ* test, showing the sensor while connected to OSA and subjected to pressure loads. The inset image shows the sensor/socket integration keeping the sensor sheet flush with the socket liner and the red colored thick arrow illustrates the vector of pressure application in perpendicular to the FBG sensor (**b**) Images depicting the setup and the heavy duty balloon used for this study.

Following the calibration, the sensor assembly was incorporated into the trans-tibial socket at the PT region (Figure 4). The alignment of the PT bar was based on the work by Abu Osman *et al.* [2] to ensure that the sensor installation was in the intended place. The sensor was placed such that it was flush with the inner socket wall for the comfort of the amputee and to reduce the risk of damage caused by the pistoning of the stump inside the socket.

An experimental setup designed to verify sensor performance *in situ* was prepared. No subjects participated in this study. Therefore, attempts were made to mimic a real-life situation. Instead of using the stump of an actual amputee, a heavy duty balloon with a thick wall was employed. Dumbleton *et al.* [4] placed a balloon in a prosthetic socket and inflated it to a known pressure, which was an effective way to calibrate stump/socket pressure sensors as well as to test sensor performance and its capability of producing repeatable patterns. As such, the balloon was inserted into the socket, to which the sensor was already attached, and connected to a pipe that was connected to an air compressor (Figure 4). The pipe contained a pressure gauge to indicate the pressure supplied to the balloon. The compressor applied cyclic air pressure into the balloon, starting from 0 psi to 5 psi (0 kPa to 34.5 kPa) and back to 0 psi to test sensor repeatability in the intended region. The sensor spectral response was recorded simultaneously.

## 3. Results and Discussion

**Figure 5.**
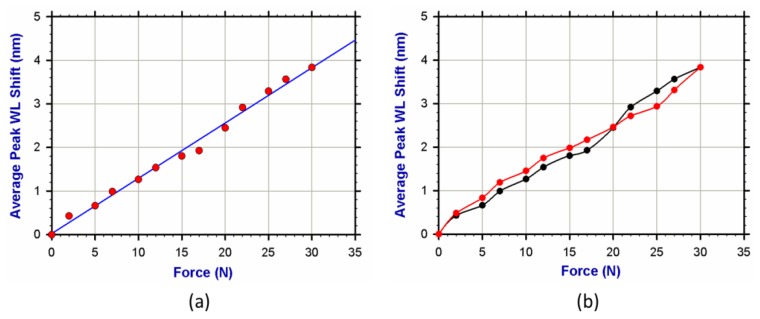
FBG sensor calibration. (**a**) The FBG peak wavelength shift *versus* the applied force and (**b**) the hysteresis error (≈0.09 FSO) shown by the sensor.

The sensor underwent seven experimental trials. The average values were used to interpret sensor behavior, which indicated that the FBG sensor was sensitive to small changes in the applied force. Figure 5a shows the calibration graph for the FBG sensor with a regression line. The sensor obviously exhibited a linear relationship between the shift in the peak wavelength and the applied force. Utilizing the slope, we calculated the sensor sensitivity at approximately 127 pm/N. From the regression line, the equation could be simply obtained as follows:

(1) Δλ=0.1269F+0.0244(nm)

where **δλ** is the wavelength shift measured in nanometers (nm), and **F** is the applied force measured in Newtons (N). Table 1 shows the specifications of the fabricated FBG sensor.

The sensor exhibited a linear response in agreement with the mechanical and optical properties of FBGs [13,14]. The line intersection with the y-axis at 0.0244 (nm) is approximately zero when compared with the full-scale output (FSO), which is about 3.8 nm (≈0.0065 of FSO). The sensor also showed a very acceptable FSO hysteresis error of around 0.09.

**Figure 6.**
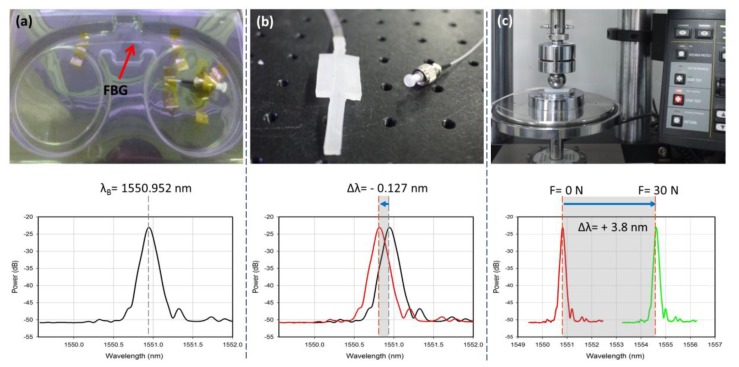
(**a**) Bare FBG spectrum. (**b**) Packaged FBG spectrum (red line) compared to the bare FBG and (**c**) Indication of the minimum (red)/maximum (green) shifts caused by the minimum/maximum applied forces during calibration.

Embedding the FBG in the host materials such as polymeric materials might cause a shift in its wavelength. The host materials upon curing may contract or expand depending on the material type. In this study, the curing process of epoxy and silicone-polymers while the FBG is embedded was monitored. It was found that the host materials contract upon curing, resulting in a slight negative shift in the FBG reflected wavelength as shown in Figure 6. This shifted wavelength was considered the initial value for the calibration.

**Figure 7.**
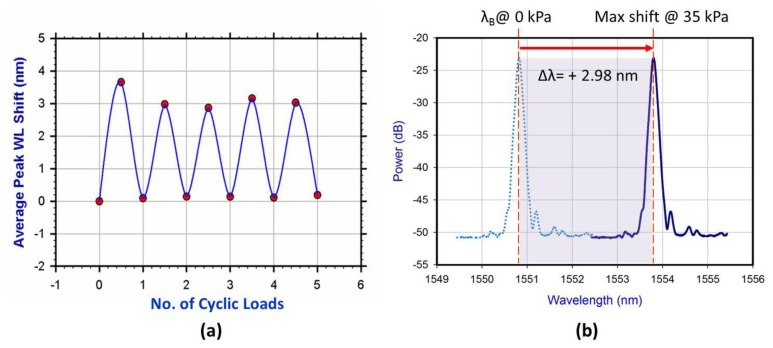
(**a**) Sensor repeatability test. Pressure was changed cyclically from minimum (0 kPa) to maximum (35 kPa) for five times to test the repeatability and reliability of the sensor. The solid line is for indicative purposes only. (**b**) The spectral response of FBG used without pressure (dotted line) and that with maximum applied pressure at ∼35 kPa (Solid line) for the second cyclic applied load.

To investigate sensor functionality at the PT region, cyclic loads were applied onto the FBG sensor to test its repeatability while it was attached to the inner socket wall. Only five load cycles were applied because the system was controlled manually. The applied maximum and minimum pressures were 35 kPa and 0 kPa, respectively. The repeatable loading pattern depicted in Figure 7a was obtained, which shows how the sensor responded to cyclic loads. The maximum and minimum wavelength values of the sinusoid-like pattern shown in Figure 7a represented the increased and decreased applied pressure, respectively. Figure 7b illustrates the wavelength shift (Δλ = 2.98 nm) resulted from applying the maximum pressure value and a zero shift when the sensor is unloaded. When the maximum pressure value, 35 kPa, was applied for the first cycle, the wavelength shift exceeded its expected value. This condition usually occurs because the OSA reading becomes constant shortly after the experiment starts. However, the outputs of the remaining cycles were very similar. These preliminary results indicate that the sensor is repeatable and capable of measuring the pressure on the socket stump interface, especially in the PT region.

This study aims to investigate the capability of FBG-based sensors to measure the interface pressure of the stump/socket of amputees. We have proposed a flexible FBG sensor configuration and design to measure the pressure on any irregularly shaped interface, such as the stump/socket interface. This study did not address pressure at other significant points within the socket wall. The temperature changes were not monitored since the experiments were conducted in a constant temperature. Involving patients as subjects to our work necessitates serious consideration of temperature variations which requires the sensor to be isolated. However, a study of these issues is in progress and involves the same concepts. We believe that this study will potentially open up an evolutionary road towards smart FBG-based amputee stump/socket structures for pressure monitoring in amputee socket systems, which will lead to a good socket fit that ensures patient satisfaction.

## 4. Conclusions

An FBG sensor was fabricated, characterized, and tested in a socket. It demonstrated very good sensitivity and acceptable hysteresis, which means that it satisfied the requirement of being able to correctly measure the pressure applied at the PT bar region. Thus, this measurement technique could provide data that contribute to the creation of improved designs for trans-tibial PTB sockets. As the polymer-embedded FBG-based sensor was very flexible, it could be expanded and attached to any irregularly shaped surface and interface. Many important areas for future work exist that allow for the successful implementation of this sensor with or without improving its composition and structure. Correlational studies are highly recommended so that the pressure profile on the PT region and other significant regions within the same trans-tibial PTB socket can be compared to create an overall picture of pressure distribution over the surface of the investigated prosthetic socket when an amputee walks. Furthermore, comparative studies should be undertaken to compare the output results of this FBG sensing method and other conventional measurement systems. A data acquisition system could be designed to be capable of measuring the pressure under dynamic conditions at the PT bar and the pressure of other significant areas within the socket wall with a frequency of up to 150 Hz. Such a system would enable researchers to monitor amputees while the latter walks on flat surfaces, slopes, and staircases or while cycling and jogging.

## Figures and Tables

**Table 1.** FBG sensor specifications.

**Specifications**	**Values**
Dynamic Range (N)	30
Max. WL Shift (nm) (During Calibration)	∼4,000
Max. WL Shift (nm) (Cyclic loads test)	∼2,980
Sensitivity (pm/N)	127
Expected error width (nm)	0.34
Hysteresis (FSO)	9%
Max FBG Disp. (mm)	1.39
Max. Power Drop (dB)	−28.9
FBG Alignment	Properly aligned
